# The correlation study between post-surgery oxygen partial pressure level and prognosis of patients with sepsis during hospitalization

**DOI:** 10.1097/MD.0000000000042449

**Published:** 2025-06-06

**Authors:** Anqi Wang, Jieying Chen, Jianling Gao

**Affiliations:** a Department of Critical Care Medicine, The Fourth Affiliated Hospital of Soochow University, Suzhou City, Jiangsu Province, China.

**Keywords:** 90-days mortality, hyperoxemia, MIMIC-IV database, restricted cubic spline, sepsis

## Abstract

Sepsis remains a leading cause of mortality and healthcare burden, necessitating improved diagnostic and therapeutic strategies. Observational studies suggest that hyperoxemia may improve postoperative sepsis outcomes, but evidence remains limited. This study aims to explore the optimal range of oxygen partial pressure (PaO2) in postsurgical sepsis patients and its impact on prognosis. Clinical data of adult sepsis patients were extracted from the medical information mart for intensive care-IV database. Patients were categorized into control (PaO2 ≤ 100 mm Hg) and hyperoxemia (PaO2 > 100 mm Hg) groups. Primary outcome was 90-day mortality, while secondary outcomes included 1-year mortality, intensive care unit (ICU)/hospital length of stay, and invasive ventilation duration. Restricted cubic spline analysis stratified postsurgical PaO2 into normal (≤128.7 mm Hg), mild hyperoxemia (128.7–162.1 mm Hg), and severe hyperoxemia (≥162.1 mm Hg). Kaplan–Meier survival analysis and multivariate regression were conducted. Among 1220 patients, hyperoxemia patients were younger, had lower disease severity, and received more aggressive treatment. They had lower rates of acute respiratory failure and acute kidney injury. After cubic spline-based classification, mild hyperoxemia was associated with lower 90-day mortality (OR: 0.54, 95% CI: 0.34–0.86, *P* = .010), while severe hyperoxemia showed no significant effect (OR: 0.60, 95% CI: 0.30–1.20, *P* = .147). Kaplan–Meier curves demonstrated significantly improved long-term survival for mild hyperoxemia patients. Mild hyperoxemia in postsurgical sepsis patients is associated with reduced 90-day mortality, suggesting a potential optimal oxygenation range for better outcomes.

## 1. Introduction

Sepsis is a life-threatening infectious disease characterized by a dysregulated host inflammatory response and multiple organ dysfunction.^[1]^ A retrospective study in China reported over 10,000 sepsis-related deaths^[2]^ and it is also one of the most costly diseases to treat.^[3]^ A meta-analysis found correlation between hyperoxemia and mortality in patients with post-surgery sepsis.^[4]^

In the management of sepsis patients, current guidelines emphasize early fluid resuscitation and timely antibiotic treatment.^[3]^ In the pathophysiological study of sepsis, some views emphasize the need to adjust the balance between oxygen delivery and consumption according to the patient’s condition. The importance of oxygen therapy is also emphasized in guidelines for the diagnosis and treatment of sepsis patients.^[3]^

Previous studies have shown that increasing the oxygen concentration and arterial partial pressure of oxygen in the blood can reduce the rate of wound infection and mortality in patients after surgery.^[5]^ However, there is currently a lack of clinical research on the role of hyperoxemia in patients with postsurgical sepsis. Some recent clinical trials have suggested that hyperoxemia is associated with an increase in 28-day and 90-day mortality.^[6–8]^ However, further research is needed to determine the optimal oxygen partial pressure range for post-surgery sepsis patients.

The aim of our retrospective study is to identify the optimal range of oxygen partial pressure for post-surgery sepsis patients and its impact on prognosis. The study aims to provide information for future clinical trials and determine the potential of hyperoxemia to improve the prognosis of post-surgery sepsis patients.

## 2. Methods

### 2.1. Data

This research uses a limited observational dataset from the Medical Information Mart for Intensive Care IV (MIMIC-IV version 2.2)^[9]^ covering the years 2008 to 2019. MIMIC-IV, a renowned publicly available critical care database, is an iteration of MIMIC-III and includes more than 50,000 intensive care unit (ICU) admissions of adult patients at the Beth Israel Deaconess Medical Centre (Boston) from 2008 to 2019. To gain access to the data, author Wang completed a certification course (certification number: 49804394), and the MIMIC-IV database protects patient privacy through anonymized personal identifiers.

### 2.2. Study group

**Figure 1.**
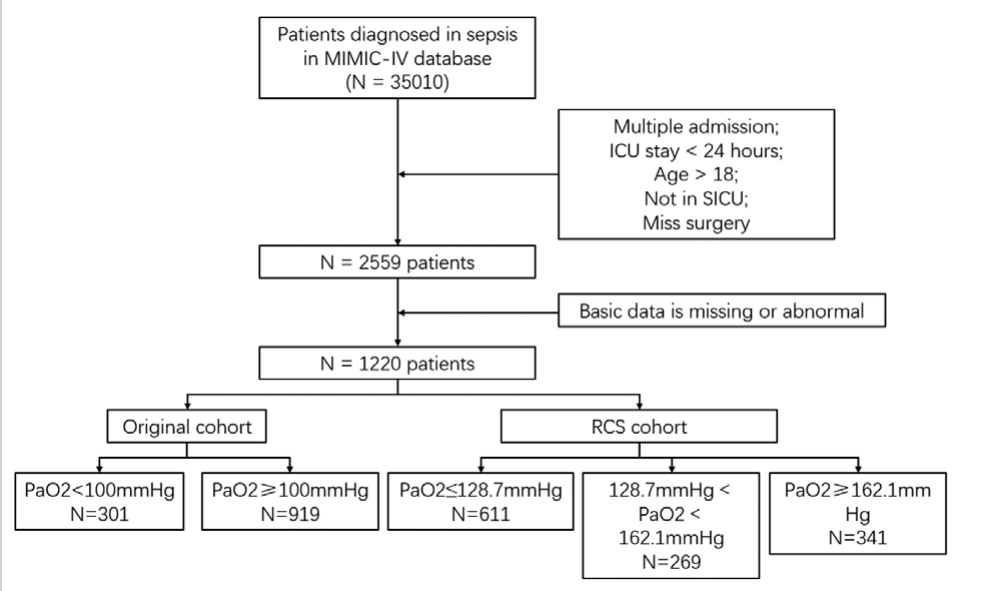
Simplified data extraction workflow for the MIMIC-IV database. MIMIC-IV = Medical Information Mart for Intensive Care IV.

All patients admitted to the ICU after surgery and meeting the Sepsis 3.0 diagnosis were screened to obtain the final group of patients in the database. The Sepsis 3.0 diagnostic criteria are: suspected bacterial infection combined with a SOFA score ≥ 2. Inclusion criteria were as follows: age over 18 years; underwent surgery; first admission to the ICU and admission to the surgical ICU; ICU stay longer than 1 day. The specific flowchart is as follows Figure 1. At the start of our study, we included patients in the hyperxemia group if their mean arterial partial pressure of oxygen (PaO2) value was >100 mmHg within 48 hours after surgery. Patients with a value less than or equal to 100 mmHg were included in the normal group. We conducted further research and performed a restricted cubic spline (RCS) analysis based on the 90-day mortality rate of patients. The overall curve showed a U-shape. Arterial oxygen partial pressure values of 128.7 mmHg and 162.1 mmHg were obtained at HR = 1, respectively. Subsequently, the subjects were grouped into 3 categories: normal group (PaO2 ≤ 128.7 mmHg), mild hyperxemia group (128.7 mmHg < PaO2 < 162.1 mmHg), and severe hyperxemia group (PaO2 ≥ 162.1 mmHg).

### 2.3. Data extraction

Data extraction was performed using structured query language (SQL) in PostgreSQL (V14.9; PostgreSQL Global Development Group). The following variables were extracted demographic characteristics such as age, gender, and vital signs such as heart rate, blood pressure, as well as the degree of organ failure within the first 24 hours of ICU admission are important predictors of patient outcome. In addition, scores such as the sequential organ failure assessment (SOFA), the simplified acute physiology score II (SAPS-II), the oxford acute severity of illness score (OASIS) and the logistic organ dysfunction score (LODS) can be used as indicators. Laboratory investigations in the first 24 hours after admission to the ICU should include tests for potassium, sodium, chloride, calcium, albumin, platelet count, white blood cell count, lymphocyte count, monocyte count, neutrophil count, PT, APTT, lactate, hemoglobin, hematocrit, creatinine, blood urea nitrogen, total bilirubin, and erythrocyte distribution width. For missing data, we used R language for screening and cleaning. For data with <20% missing, we used multiple imputation (n = 5) to supplement the data. For data with 20% or more missing, we excluded the test data from this study. Relevant treatment details (e.g. whether invasive ventilation or renal replacement therapy was used) should also be provided. For variables with multiple measurements during the inclusion period (e.g. heart rate), the mean was calculated. For multiple assessments of patient condition scores (e.g. SAPS-II, SOFA, OASIS, LODS), only the highest score was used.

### 2.4. Clinical outcomes

The primary clinical outcome of this study was patient mortality within 90 days. The secondary outcomesincluded 1-year mortality, ICU and hospital length of stay.

### 2.5. Statistical analysis

First, univariate analysis was performed to compare all variables in the study. Mean and standard deviation were used to describe continuous variables and paired t-test was used for comparison. Categorical variables were presented as numbers and percentages and compared using Fisher exact test where appropriate. The probability of death for both groups of patients at 28, 90, 180, and 365 days after ICU admission was analyzed using Kaplan–Meier survival curves. Variables with a *P*-value < .1 in the univariate regression analysis were considered as adjustment variables in the multivariate analysis. R version 4.3.2 was used for all data analyses.

## 3. Results

### 3.1. Demographic and underlying data characteristics

This retrospective study analyzed data from 1220 patients who met the Sepsis 3.0 diagnostic criteria after undergoing surgery, using the MIMIC public database. Of these patients, 301 had an average oxygen partial pressure within the normal range within 48 hours of surgery, while 919 developed hyperoxaemia postoperatively. Table 1 presents baseline data, first-day laboratory indicators, and first-day vital signs of all patients included in the study after admission to the ICU. The mean age of patients with hyperoxemia (59.23 ± 17.88) was lower than that of patients with normal oxygen partial pressure (61.63 ± 16.82). When comparing the admission PaO2 levels between the 2 groups, it was found that the patients with normal oxygen partial pressure had levels closer to the normal range (113.53 ± 42.16 vs 164.44 ± 62.03, *P* < .001). Patients with hyperoxemia had a less severe assessment of disease compared to patients with normal partial pressure of oxygen. This was evidenced by lower 90-day mortality rates (23.72% vs 32.23%), lower SOFA scores (6.28 ± 3.35 vs 6.85 ± 3.62), lower Charlson comorbidity index (5.04 ± 3.05 vs 5.44 ± 2.82), and lower LODS (7.08 ± 3.26 vs 8.03 ± 3.29). The admission conditions showed a lower proportion of patients with hyperoxemia who had acute renal failure (43.31% vs 53.82%) and acute respiratory failure (53.65% vs 68.77%). No significant differences were found in other covariates (*P* > .05).

**Table 1:** Comparisons of baseline characteristics between Normoxemia and Hyperoxemia.

Variables	Total	Normoxemia	Hyperoxemia	*P*-value
	(n = 1220)	(n = 301)	1 (n = 919)	
Age (yr)	59.82 ± 17.65	61.63 ± 16.82	59.23 ± 17.88	.041
Height (cm)	169.66 ± 11.89	169.34 ± 10.97	169.76 ± 12.18	.588
Weight (kg)	86.33 ± 26.30	88.91 ± 28.82	85.48 ± 25.38	.066
BMI, mean ± SD	33.49 ± 123.50	31.12 ± 10.15	34.26 ± 142.18	.702
90-d morality, n (%)	315 (25.82)	97 (32.23)	218 (23.72)	.003
Gender = M, n (%)	739 (60.57)	184 (61.13)	555 (60.39)	.820
Los Hospital, mean ± SD	22.33 ± 18.88	22.07 ± 17.14	22.42 ± 19.43	.782
Los ICU, mean ± SD	13.34 ± 11.90	15.28 ± 13.63	12.70 ± 11.22	.003
First-day laboratory examination				
Sodium (mEq/L)	139.01 ± 4.02	138.54 ± 4.33	139.17 ± 3.90	.027
Potassium (mEq/L)	4.13 ± 0.50	4.14 ± 0.50	4.13 ± 0.51	.733
Chloride (mEq/L)	105.97 ± 5.20	104.57 ± 5.63	106.43 ± 4.96	<.001
Calcium (mg/dL)	8.13 ± 0.76	8.14 ± 0.75	8.13 ± 0.76	.884
Platelet (K/µL)	213.06 ± 114.21	219.28 ± 122.14	211.03 ± 111.48	.277
WBC (K/µL)	13.04 ± 6.90	13.39 ± 7.54	12.92 ± 6.68	.308
RBC (K/µL)	3.64 ± 0.67	3.64 ± 0.75	3.64 ± 0.64	.887
PT (s)	15.49 ± 4.93	15.83 ± 5.15	15.37 ± 4.86	.168
PTT (s)	34.12 ± 10.74	34.54 ± 12.06	33.98 ± 10.27	.431
INR	1.40 ± 0.47	1.43 ± 0.49	1.38 ± 0.46	.124
PH	7.38 ± 0.06	7.38 ± 0.06	7.38 ± 0.05	.694
PCO2 (mm Hg)	40.37 ± 6.29	41.75 ± 6.97	39.92 ± 5.98	<.001
PO2 (mm Hg)	151.88 ± 61.79	113.53 ± 42.16	164.44 ± 62.03	<.001
Hemoglobin (g/dL)	10.93 ± 1.96	10.95 ± 2.13	10.92 ± 1.90	.840
Hematocrit (%)	32.61 ± 5.73	33.13 ± 6.39	32.44 ± 5.49	.094
MCH (pg)	30.17 ± 2.36	30.22 ± 2.48	30.16 ± 2.32	.686
MCHC (%)	33.44 ± 1.63	33.02 ± 1.72	33.57 ± 1.58	<.001
MCV (fL)	90.35 ± 6.52	91.61 ± 6.51	89.94 ± 6.47	<.001
Creatinine (mg/dL)	1.33 ± 1.20	1.37 ± 1.33	1.31 ± 1.15	.459
BUN (mg/dL)	24.42 ± 18.52	25.53 ± 17.65	24.06 ± 18.79	.234
RDW (%)	15.01 ± 1.96	15.19 ± 2.18	14.95 ± 1.88	.092
Severity of organ dysfunction				
Charlson comorbidity	5.14 ± 3.00	5.44 ± 2.82	5.04 ± 3.05	.039
SAPS-II	41.64 ± 14.72	42.86 ± 15.64	41.24 ± 14.39	.098
SOFA	6.42 ± 3.43	6.85 ± 3.62	6.28 ± 3.35	.012
OASIS	39.93 ± 8.69	40.57 ± 8.78	39.72 ± 8.65	.141
LODS	7.32 ± 3.29	8.03 ± 3.29	7.08 ± 3.26	<.001
First-day vitalsign				
Heart rate (bpm)	90.66 ± 17.46	92.78 ± 18.27	89.97 ± 17.15	.015
SBP (mm Hg)	117.97 ± 15.91	116.80 ± 16.22	118.35 ± 15.79	.140
DBP (mm Hg)	62.20 ± 9.99	62.35 ± 10.38	62.15 ± 9.87	.761
MBP (mm Hg)	78.88 ± 10.41	77.97 ± 10.80	79.18 ± 10.26	.081
Resp rate (bpm)	19.64 ± 4.05	20.63 ± 4.47	19.32 ± 3.84	<.001
Temperature (°C)	37.06 ± 0.63	37.07 ± 0.63	37.05 ± 0.64	.631
Spo2, n (%)	97.57 ± 2.10	96.46 ± 2.18	97.93 ± 1.94	<.001
Glucose (mg/dL)	147.00 ± 40.05	144.86 ± 46.13	147.70 ± 37.85	.336
surgery_po2_48h (mm Hg)	141.00 ± 55.23	84.80 ± 10.94	159.41 ± 51.35	<.001
Fio2, n (%)	36.14 ± 21.54	34.72 ± 20.23	36.61 ± 21.95	.188
PaO2/FiO2, mean ± SD	500.28 ± 291.15	307.04 ± 123.85	563.58 ± 302.15	<.001
IMV, n(%)				.188
No	5 (0.41)	3 (1.00)	2 (0.22)	
Yes	1215 (99.59)	298 (99.00)	917 (99.78)	
RRT, n(%)				.091
No	1064 (87.21)	254 (84.39)	810 (88.14)	
Yes	156 (12.79)	47 (15.61)	109 (11.86)	
Vasoactive, n(%)				
No	423 (34.67)	95 (31.56)	328 (35.69)	.191
Yes	797 (65.33)	206 (68.44)	591 (64.31)	
Ventilation status				
Invasivevent, n (%)				
No	120 (9.84)	33 (10.96)	87 (9.47)	.449
Yes	1100 (90.16)	268 (89.04)	832 (90.53)	
Oxygen, n (%)				
No	323 (26.48)	90 (29.90)	233 (25.35)	.121
Yes	897 (73.52)	211 (70.10)	686 (74.65)	
Noninvasivevent, n (%)				
No	1191 (97.62)	290 (96.35)	901 (98.04)	.094
Yes	29 (2.38)	11 (3.65)	18 (1.96)	
Highflow, n (%)				
No	1109 (90.90)	259 (86.05)	850 (92.49)	<.001
Yes	111 (9.10)	42 (13.95)	69 (7.51)	
Trach, n (%)				
No	1136 (93.11)	264 (87.71)	872 (94.89)	<.001
Yes	84 (6.89)	37 (12.29)	47 (5.11)	
Commorbity				
AMI, n (%)				
No	1209 (99.10)	299 (99.34)	910 (99.02)	.881
Yes	11 (0.90)	2 (0.66)	9 (0.98)	
Coronary Atherosclerosis, n (%)				
No	1004 (82.30)	250 (83.06)	754 (82.05)	.690
Yes	216 (17.70)	51 (16.94)	165 (17.95)	
Valve disorder, n (%)				
No	1143 (93.69)	284 (94.35)	859 (93.47)	.585
Yes	77 (6.31)	17 (5.65)	60 (6.53)	
AKI, n(%)				
No	660 (54.10)	139 (46.18)	521 (56.69)	.001
Yes	560 (45.90)	162 (53.82)	398 (43.31)	
ARF, n(%)				
No	520 (42.62)	94 (31.23)	426 (46.35)	<.001
Yes	700 (57.38)	207 (68.77)	493 (53.65)	
Diabete, n(%)				
No	829 (67.95)	192 (63.79)	637 (69.31)	.075
Yes	391 (32.05)	109 (36.21)	282 (30.69)	
Hypertension, n(%)				
No	492 (40.33)	112 (37.21)	380 (41.35)	.204
Yes	728 (59.67)	189 (62.79)	539 (58.65)	

AKI = acute kidney injury, AMI = acute myocardial infarction, ARF = acute respiratory failure, LODS = logistic organ insufficiency, MAP = mean arterial pressure, OASIS = Oxford acute disease severity, SAPS-II = simplified acute physiology score II, SOFA = sequential organ failure assessment, SpO2 = peripheral capillary oxygen saturation, VD = valve disorder, ventilation status = The condition that the patient has received corresponding oxygen therapy in this study.

## 4. The association between post-surgery hyperxemia and mortality outcomes

**Figure 2.**
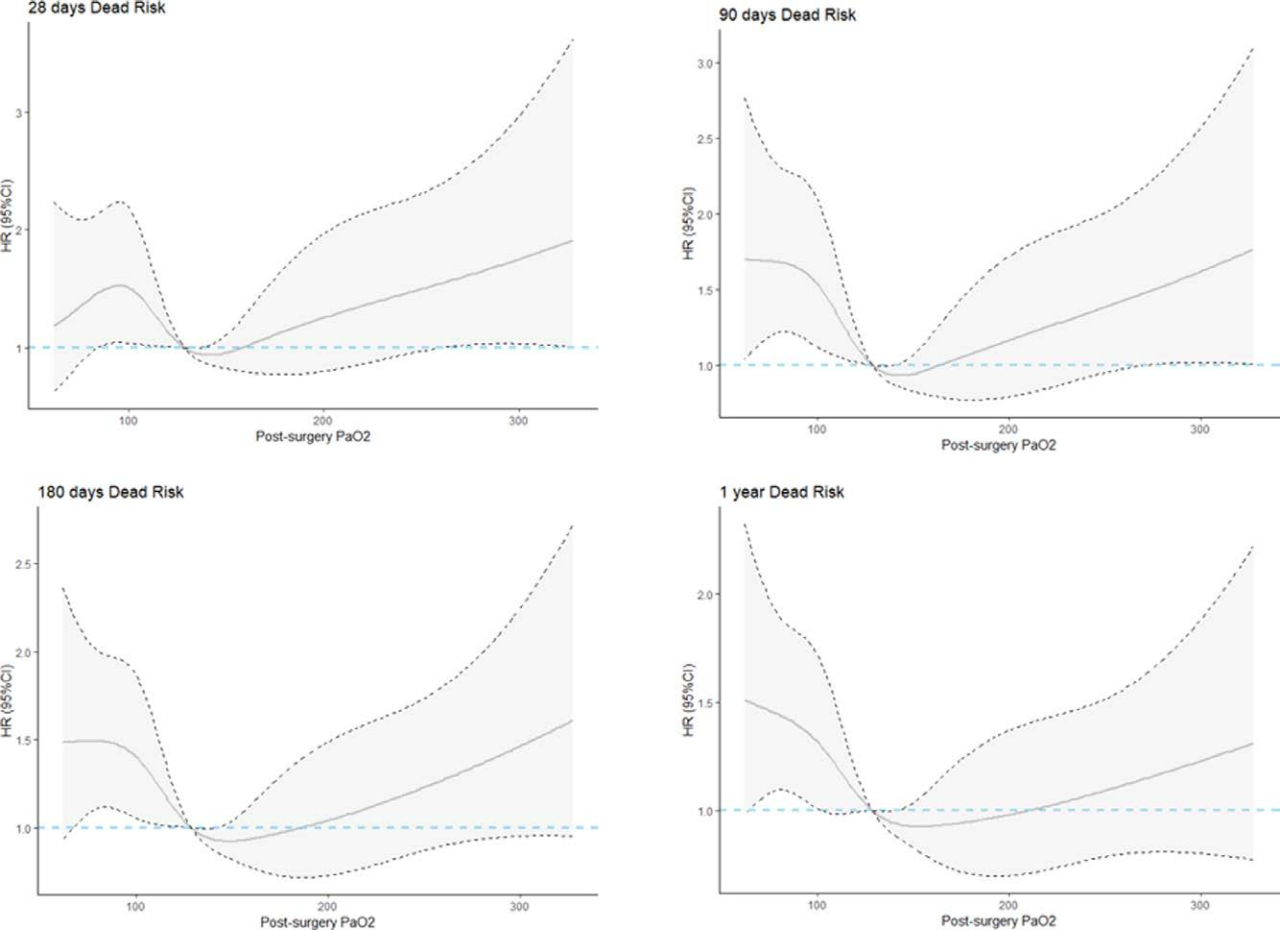
Restricted cubic splines were calculated based on the mortality rates at 28 d, 90 d, 180 d, and 1 yr.

The RCS model in this study reveals a U-shaped association between post-surgeryarterial oxygen partial pressure levels and all-cause mortality, as demonstrated in Figure 2. The patients were divided into 3 groups based on their PaO2 value at HR = 1 in the study results of the 90-day mortality rate in the RCS: the normal oxygen partial pressure group (PaO2 ≤ 128.7 mmHg), the mild hyperoxemia group (128.7 mmHg < PaO2 < 162.1 mmHg), and the severe hyperoxemia group (PaO2 ≥ 162.1 mmHg). After regrouping, we reconstructed Table 2 with the patient’s baseline data. The study shows that the mortality rate is lower (*P* < .05) when the patient’s oxygen partial pressure is between 128.7 mmHg and 162.1 mmHg, as calculated by RCS, compared to the other 2 groups.

**Table 2:** Basic data table after grouping using restricted cubic splines.

Variables	Total	Normoxemia	Mild hyperoxemia	Severe hyperoxemia	P-Value
	(n = 1220)	(n = 611)	(n = 269)	(n = 340)	
Age (yr)	59.82 ± 17.65	61.57 ± 16.75	61.33 ± 16.87	**55.49 ± 19.09**	**<.001**
Height (cm)	169.66 ± 11.89	170.15 ± 10.87	169.26 ± 14.86	**169.10 ± 10.98**	**.353**
Weight (kg)	86.33 ± 26.30	89.63 ± 28.47	85.15 ± 20.84	**81.32 ± 25.29**	**<.001**
Bmi, Mean ± SD	33.49 ± 123.50	30.89 ± 9.39	45.77 ± 262.45	**28.42 ± 8.43**	**.173**
90-days morality, n (%)	315 (25.82)	181 (29.62)	51 (18.96)	**83 (24.41**)	**.003**
Los Hospital, Mean ± SD	22.33 ± 18.88	23.14 ± 18.73	21.38 ± 17.60	**21.62 ± 20.10**	**.32**
Los Icu, Mean ± SD	13.34 ± 11.90	15.35 ± 13.18	11.89 ± 9.72	**10.87 ± 10.35**	**<.001**
First-day laboratory examination					
Sodium (mEq/L)	139.01 ± 4.02	138.72 ± 4.24	139.05 ± 3.72	**139.51 ± 3.79**	**.013**
Potassium (mEq/L)	4.13 ± 0.50	4.15 ± 0.53	4.09 ± 0.47	**4.12 ± 0.48**	**.210**
Chloride (mEq/L)	105.97 ± 5.20	105.18 ± 5.54	106.20 ± 4.48	**107.22 ± 4.83**	**<.001**
Calcium (mg/dL)	8.13 ± 0.76	8.10 ± 0.76	8.13 ± 0.68	**8.19 ± 0.81**	**.218**
Platelet (K/µL)	213.06 ± 114.21	213.94 ± 108.88	210.46 ± 127.38	**213.56 ± 112.85**	**.913**
WBC (K/µL)	13.04 ± 6.90	13.46 ± 7.49	12.70 ± 6.28	**12.55 ± 6.20**	**.094**
RBC (K/µL)	3.64 ± 0.67	3.65 ± 0.71	3.69 ± 0.62	**3.58 ± 0.63**	**.106**
PT (s)	15.49 ± 4.93	15.87 ± 5.28	15.13 ± 4.07	**15.07 ± 4.87**	**.022**
PTT (s)	34.12 ± 10.74	34.24 ± 11.18	33.69 ± 10.39	**34.24 ± 10.21**	**.761**
INR	1.40 ± 0.47	1.43 ± 0.51	1.37 ± 0.41	**1.35 ± 0.43**	**.021**
PH	7.38 ± 0.06	7.37 ± 0.06	7.38 ± 0.06	**7.38 ± 0.05**	**.031**
PCO2 (mm Hg)	40.37 ± 6.29	41.32 ± 6.63	40.06 ± 5.86	**38.91 ± 5.65**	**<.001**
PO2 (mm Hg)	151.88 ± 61.79	120.90 ± 39.42	151.19 ± 50.30	**208.10 ± 63.50**	**<.001**
Hemoglobin (g/dL)	10.93 ± 1.96	10.93 ± 2.08	11.11 ± 1.92	**10.78 ± 1.76**	**.107**
Hematocrit (%)	32.61 ± 5.73	32.90 ± 6.14	33.03 ± 5.48	**31.78 ± 5.06**	**.006**
MCH (pg)	30.17 ± 2.36	30.08 ± 2.43	30.23 ± 2.26	**30.29 ± 2.31**	**.359**
MCHC (%)	33.44 ± 1.63	33.18 ± 1.61	33.48 ± 1.62	**33.87 ± 1.58**	**<.001**
MCV (fL)	90.35 ± 6.52	90.73 ± 6.48	90.47 ± 6.41	**89.59 ± 6.62**	**.034**
Creatinine (mg/dL)	1.33 ± 1.20	1.38 ± 1.23	1.39 ± 1.20	**1.20 ± 1.13**	**.060**
BUN (mg/dL)	24.42 ± 18.52	26.39 ± 19.28	24.91 ± 18.79	**20.51 ± 16.20**	**<.001**
RDW (%)	15.01 ± 1.96	15.07 ± 1.99	14.98 ± 1.85	**14.93 ± 1.98**	**.581**
Severity of organ dysfunction					
Charlson comorbidity	5.14 ± 3.00	5.33 ± 2.87	5.35 ± 2.94	**4.63 ± 3.23**	**.001**
SAPS-II	41.64 ± 14.72	42.45 ± 14.69	41.53 ± 14.32	**40.26 ± 15.01**	**.088**
SOFA	6.42 ± 3.43	6.83 ± 3.57	6.41 ± 3.25	**5.69 ± 3.19**	**<.001**
OASIS	39.93 ± 8.69	40.76 ± 8.82	39.11 ± 8.74	**39.09 ± 8.27**	**.004**
LODS	7.32 ± 3.29	7.91 ± 3.29	6.96 ± 3.26	**6.54 ± 3.13**	**<.001**
First-day vitalsign					
Heart rate (bpm)	90.66 ± 17.46	91.20 ± 17.98	88.84 ± 15.99	**91.13 ± 17.59**	**.153**
SBP (mm Hg)	117.97 ± 15.91	117.29 ± 16.10	118.37 ± 16.18	**118.86 ± 15.32**	**.310**
DBP (mm Hg)	62.20 ± 9.99	61.92 ± 10.16	61.37 ± 9.51	**63.34 ± 9.98**	**.033**
MBP (mm Hg)	78.88 ± 10.41	77.88 ± 10.25	78.90 ± 10.48	**80.67 ± 10.41**	**<.001**
Resp rate (bpm)	19.64 ± 4.05	20.37 ± 4.23	19.28 ± 3.68	**18.61 ± 3.71**	**<.001**
Temperature (℃)	37.06 ± 0.63	37.05 ± 0.65	37.10 ± 0.63	**37.04 ± 0.61**	**.422**
Spo2, n (%)	97.57 ± 2.10	96.94 ± 2.06	97.72 ± 2.23	**98.58 ± 1.60**	**<.001**
Glucose(mg/dL)	147.00 ± 40.05	146.60 ± 43.63	147.59 ± 36.46	**147.24 ± 35.93**	**.935**
surgery_po2_48 h (mm Hg)	141.00 ± 55.23	99.79 ± 17.57	143.62 ± 9.25	**212.98 ± 45.64**	**<.001**
Fio2, n(%)	36.14 ± 21.54	35.78 ± 21.00	35.90 ± 21.08	**36.98 ± 22.86**	**.701**
PaO2/FiO2, Mean ± SD	500.28 ± 291.15	355.16 ± 156.15	511.09 ± 208.19	**752.53 ± 353.27**	**<.001**
Gender, n (%)					**.085**
F	481 (39.43)	228 (37.32)	102 (37.92)	**151 (44.41**)	
M	739 (60.57)	383 (62.68)	167 (62.08)	**189 (55.59**)	
RRT, n (%)					**.003**
NO	1064 (87.21)	513 (83.96)	243 (90.33)	**308 (90.59**)	
YES	156 (12.79)	98 (16.04)	26 (9.67)	**32 (9.41**)	
Vasoactive, n(%)					**.095**
NO	423 (34.67)	197 (32.24)	107 (39.78)	**119 (35.00**)	
YES	797 (65.33)	414 (67.76)	162 (60.22)	**221 (65.00**)	
Ventilation status					
Invasivevent, n (%)					**.689**
NO	120 (9.84)	59 (9.66)	30 (11.15)	**31 (9.12**)	
YES	1100 (90.16)	552 (90.34)	239 (88.85)	**309 (90.88**)	
Oxygen, n (%)					**.108**
NO	323 (26.48)	173 (28.31)	58 (21.56)	**92 (27.06**)	
YES	897 (73.52)	438 (71.69)	211 (78.44)	**248 (72.94**)	
Noninvasivevent, n (%)					**.353**
NO	1191 (97.62)	593 (97.05)	263 (97.77)	**335 (98.53**)	
YES	29 (2.38)	18 (2.95)	6 (2.23)	**5 (1.47**)	
Highflow, n (%)					**.009**
NO	1109 (90.90)	541 (88.54)	247 (91.82)	**321 (94.41**)	
YES	111 (9.10)	70 (11.46)	22 (8.18)	**19 (5.59**)	
Trach, n (%)					**<.001**
NO	1136 (93.11)	547 (89.53)	254 (94.42)	**335 (98.53**)	
YES	84 (6.89)	64 (10.47)	15 (5.58)	**5 (1.47**)	
Commorbity					
AMI, n (%)					**.790**
NO	1209 (99.10)	605 (99.02)	266 (98.88)	**338 (99.41**)	
YES	11 (0.90)	6 (0.98)	3 (1.12)	**2 (0.59**)	
Coronary atherosclerosis, n (%)					**<.001**
NO	1004 (82.30)	500 (81.83)	202 (75.09)	**302 (88.82**)	
YES	216 (17.70)	111 (18.17)	67 (24.91)	**38 (11.18**)	
Valve disorder, n (%)					**.356**
NO	1143 (93.69)	575 (94.11)	247 (91.82)	**321 (94.41**)	
YES	77 (6.31)	36 (5.89)	22 (8.18)	**19 (5.59**)	
AKI, n (%)					**<.001**
NO	660 (54.10)	293 (47.95)	151 (56.13)	**216 (63.53**)	
YES	560 (45.90)	318 (52.05)	118 (43.87)	**124 (36.47**)	
ARF, n (%)					**<.001**
NO	520 (42.62)	200 (32.73)	136 (50.56)	**184 (54.12**)	
YES	700 (57.38)	411 (67.27)	133 (49.44)	**156 (45.88**)	
Diabete, n (%)					**<.001**
NO	829 (67.95)	388 (63.50)	176 (65.43)	**265 (77.94**)	
YES	391 (32.05)	223 (36.50)	93 (34.57)	**75 (22.06**)	
Hypertension, n (%)					**.010**
NO	492 (40.33)	241 (39.44)	93 (34.57)	**158 (46.47**)	
YES	728 (59.67)	370 (60.56)	176 (65.43)	**182 (53.53**)	

AKI = acute kidney injury, AMI = acute myocardial infarction, ARF = acute respiratory failure, LODS = logistic organ insufficiency, MAP = mean arterial pressure, OASIS = Oxford acute disease severity, SAPS-II = simplified acute physiology score II, SOFA = sequential organ failure assessment, SpO2 = peripheral capillary oxygen saturation, VD = valve disorder, Ventilation Status = The condition that the patient has received corresponding oxygen therapy in this study.

The patient’s baseline data was recreated based on the PaO2 value taken in the RCS model of their 90-day mortality rate. Table 2 shows that 611 people had a post-surgeryoxygen partial pressure <128.7 mmHg, 269 people were in the mild hyperoxemia group, and 340 people were in the severe hyperoxemia group. The study cohort reveals that patients with mild and severe hyperoxemia are younger than the normal group and have a lower 90-day mortality rate. Additionally, they exhibit similar disease severity scores, including lower SOFA, OASIS, and LODS scores. Regarding admission conditions, patients with mild and severe hyperoxemia exhibited similar statistical significance in relation to acute respiratory failure, acute renal failure, diabetes, hypertension, and other conditions (*P* < .01).

**Figure 3.**
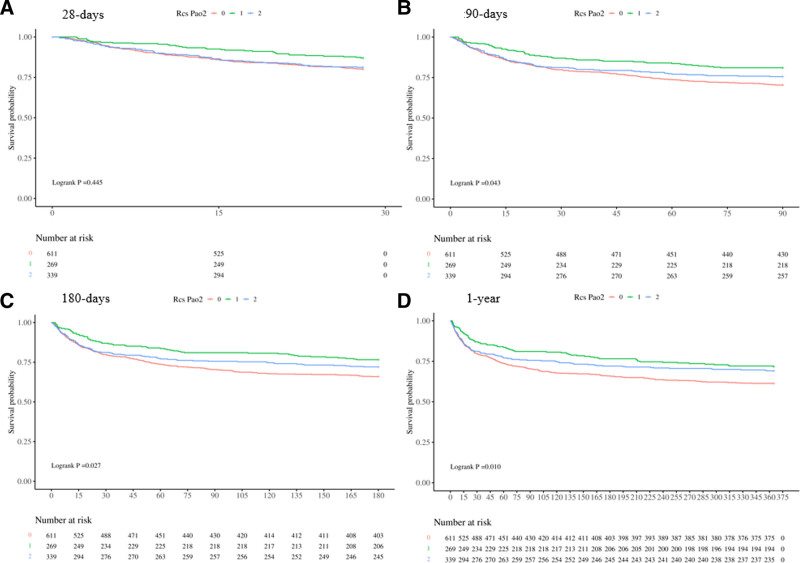
Kaplan–Meier survival curves at 28 d, 90 d, 180 d, and 1 yr plotted after grouping based on RCS curves. RCS = restricted cubic spline.

To determine whether mild to severe hyperxemia, based on RCS grouping, is an independent risk factor for increased all-cause mortality in postoperative patients, we conducted univariate and multivariate Cox regression analyses (Table 3). The results of the univariate model showed a close relationship between mild (OR: 0.56; HR: 0.39–0.79, *P* = .001) after RCS analysis and 90-day mortality (Fig. 3). After adjusting for other confounding factors in the multivariate model, there remained a significant correlation(OR: 0.54; HR: 0.34–0.86, *P* = .01) between mild hyperxemia patients and 90-day mortality after RCS analysis. However, no significant statistical significance was found in severe hyperxemia patients.

## 5. Subgroup analysis and forest mapping

**Figure 4.**
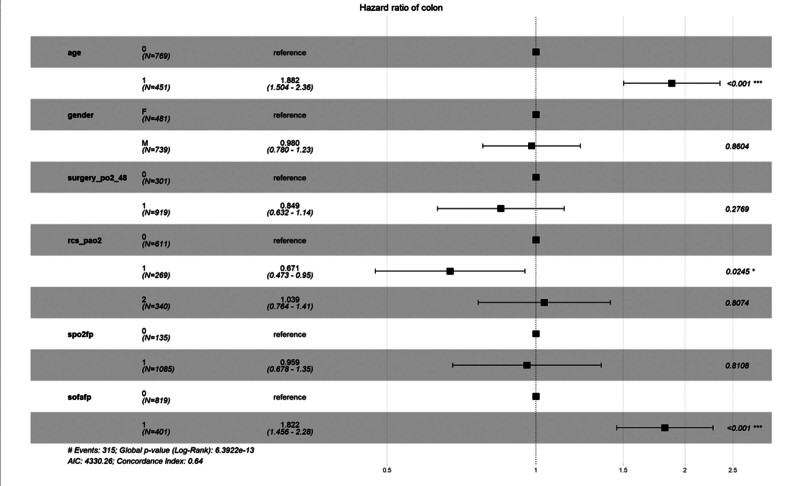
Forest plot for subgroup analysis of the relationship between 90-d mortality rate after ICU admission and oxygen partial pressure levels after RCS grouping. sofafp：0：SOFA＜7；1：SOFA ≥ 7. spo2fp：0：SpO2＜95%；1：SpO2 ≥ 95%. Surgery_po2_48:0: post-surgery pao2 < 100 mmhg;1:post-surgery pao2 ≥ 100 mmHg. rcs_pao2:0: post-surgery pao2 ≤ 128.7 mmHg; 1: 128.7 mmHg < post-surgery pao2 < 162.1 mmHg; 2: post-surgery pao2 ≥ 162.1 mmHg. ICU = intensive care unit, RCS = restricted cubic spline.

Figure 4 shows that after grouping using RCS, the correlation between the level of oxygen partial pressure and the 90-day all-cause mortality rate in patients with sepsis after surgery remains stable in all subgroups. We conducted a stratified analysis forest plot for age, gender, SOFA score, blood oxygen saturation, and oxygen partial pressure grouped using traditional methods. The results show that except for age and SOFA score, the level of oxygen partial pressure grouped by RCS is an independent influencing factor.

## 6. Discussion

The study results indicate that patients with oxygen partial pressure between 128.6 mmHg and 162.1 mmHg within 48 hours after surgery have a longer survival time compared to those in the higher and lower PaO2 groups. This is a significant factor in the all-cause mortality rate of patients admitted to the ICU for 90 days, 180 days, and 1 year. However, there is no statistical significance in the 28-day mortality rate. A nonlinear relationship was found between post-surgeryPaO2 and all-cause mortality. The risk of all-cause death significantly increases when PaO2 is <128.6 mmHg or >162.1 mmHg. Subsequent KM survival curves and Cox regression analysis further confirmed that the all-cause mortality rate of patients with oxygen partial pressure within the range of 128.6 mmHg to 162.1 mmHg is higher than the other 2 groups, and this phenomenon still exists in subgroup analysis. In summary, this study demonstrates that maintaining appropriate higher PaO2 levels after surgery has a positive impact on the long-term prognosis of patients with post-surgery sepsis. This finding can aid in the development of more effective clinical treatment plans for sepsis patients.

Although previous studies^[10–13]^ have presented conflicting evidence on the effect of hyperoxemia on the prognosis of patients with sepsis and those undergoing surgery, it is confirmed that providing appropriate respiratory support for sepsis patients is necessary.^[14]^ As sepsis is a complex clinical phenomenon, we defined hyperoxemia as a PaO2 level greater than or equal to 100 mmHg at the beginning of the study. We then used RCS to calculate the appropriate PaO2 range in subsequent studies. Our retrospective analysis found that maintaining a slightly higher than normal level of PaO2 after surgery can improve long-term survival rates. This finding has broad implications for the management and prognosis of patients with sepsis.

In this retrospective study, although we statistically analyzed the oxygen therapy methods for all patients, there is no good way to confirm the cause of hyperoxaemia in postsurgical sepsis patients. For a long time, oxygen inhalation has been considered the cornerstone of sepsis treatment,^[14]^ and previous animal studies have suggested that hyperoxia treatment can alleviate organ damage caused by sepsis.^[15]^ However, some literature suggests that hyperoxia may increase tissue oxygen tension, which has adverse effects on organ perfusion and microcirculatory blood flow.^[16,17]^ Oxygen, as one of the strongest oxidants, can induce excessive production of reactive oxygen species (ROS) in a high oxygen environment,^[18]^ and cause oxidative stress-induced damage in subsequent disease progression. This is particularly evident in patients with septic shock. Reports suggest that^[19]^ impaired mitochondrial respiration and weakened antioxidant defences may increase ROS production and further exacerbate the manifestations of injury. In addition, there are several reports indicating that the mortality rate of patients with hyperoxemia is significantly increased.^[20–22]^ On the other hand, hyperoxemia also plays a beneficial role in patient prognosis. Theoretically, hyperoxemia may enhance the ability of vasoconstriction to prevent hypotension,^[23]^ and O2 has certain antibacterial effects.^[5]^ Based on this, studies suggest that the possible reason for hyperoxemia to improve tissue organ function is that it can induce systemic vasoconstriction and compensate for SIRS-induced vascular paralysis, thereby redistributing circulating blood to vital organs.^[24]^ At the same time, some clinical studies claim that hyperoxemia can reduce wound infection in perioperative patients,^[5]^ shorten hospital stay and extubation time.^[6]^ However, more research is needed to confirm which factors mediate the effect of hyperoxemia on long-term patient prognosis.

## 7. Limitation

### 7.1. Retrospective design and causality

The retrospective nature of this study introduces potential selection bias and limits the ability to establish a causal relationship between postoperative PaO2 levels and mortality. Despite statistical adjustments, unmeasured confounders may have influenced the results. Future prospective studies, particularly randomized controlled trials, are needed to validate these findings.

### 7.2. Lack of mechanistic explanation

While the study identifies a nonlinear relationship between PaO2 and mortality, it does not provide direct mechanistic evidence to explain this association. The role of oxidative stress, microcirculatory dysfunction and tissue oxygenation imbalance has been hypothesized but not experimentally confirmed. Further research using molecular and physiological markers is needed to clarify these mechanisms.

### 7.3. Variability in oxygen therapy and patient factors

The study accounts for variations in oxygen therapy, but does not fully account for differences in ventilation strategies, patient conditions and individual responses to oxygen levels. These factors may influence the results and limit the generalisability of the findings. Standardised protocols for oxygen administration should be considered in future research to reduce variability and improve clinical applicability.

## 8. Conclusion

In conclusion, sepsis patients with postoperative hyperoxaemia show a significant reduction in 90-day mortality with comparable long-term prognosis. However, hyperoxaemic patients did not show a significant improvement in length of hospital stay or ICU admission. Based on these findings, clinicians should carefully balance oxygen therapy to optimize survival benefits while minimizing potential adverse effects. Future research should focus on identifying the precise mechanisms underlying the impact of hyperoxemia on sepsis prognosis and determining individualized oxygen therapy strategies to improve patient outcomes.

**Table 3:** Univariate and multivariate analysis for evaluating the risk of mortality at 90 d.

Dependent: 90-d-mortality		DEAD (N = 905)	SURVIVE (N = 315)	OR (univariable)	OR (multivariable)
RRT	NO	812 (89.7%)	252 (80%)		
	YES	93 (10.3%)	63 (20%)	2.18 (1.54–3.10, *P* < .001)	1.28 (0.80–2.04, *P* = .303)
Vasoactive	NO	345 (38.1%)	78 (24.8%)		
	YES	560 (61.9%)	237 (75.2%)	1.87 (1.40–2.50, *P* < .001)	1.23 (0.86–1.74, *P* = .254)
Charlson	Mean ± SD	4.6 ± 2.9	6.6 ± 2.9	1.26 (1.20–1.32, *P* < .001)	1.24 (1.17–1.31, *P* < .001)
SAPS-II	Mean ± SD	39.4 ± 13.8	48.2 ± 15.3	1.04 (1.03–1.05, *P* < .001)	1.00 (0.99–1.02, *P* = .630)
SOFA	Mean ± SD	6.1 ± 3.2	7.5 ± 3.8	1.12 (1.08–1.17, *P* < .001)	1.00 (0.94–1.07, *P* = .958)
OASIS	Mean ± SD	38.9 ± 8.5	42.8 ± 8.5	1.05 (1.04–1.07, *P* < .001)	1.01 (0.99–1.03, *P* = .483)
LODS	Mean ± SD	6.8 ± 3.2	8.8 ± 3.1	1.20 (1.15–1.25, *P* < .001)	1.17 (1.09–1.26, *P* < .001)
Glucose	Mean ± SD	145.8 ± 38.1	150.6 ± 45.1	1.00 (1.00–1.01, *P* = .068)	1.00 (1.00–1.00, *P* = .551)
Postsurgical PaO2	Mean ± SD	142.0 ± 53.7	138.0 ± 59.5	1.00 (1.00–1.00, *P* = .263)	1.00 (1.00–1.01, *P* = .231)
Fio2	Mean ± SD	35.6 ± 21.0	37.7 ± 23.1	1.00 (1.00–1.01, *P* = .131)	
PaO2/FiO2	Mean ± SD	507.2 ± 284.1	480.4 ± 310.2	1.00 (1.00–1.00, *P* = .160)	1.00 (1.00–1.00, *P* = .327)
Los_hospital	Mean ± SD	23.8 ± 19.9	18.2 ± 14.9	0.98 (0.97–0.99, *P* < .001)	0.94 (0.92–0.96, *P* < .001)
Los_icu	Mean ± SD	13.4 ± 11.9	13.3 ± 11.9	1.00 (0.99–1.01, *P* = .892)	
RCS_Pao2	Normoxemia	430 (47.5%)	181 (57.5%)		
	Mild hyperoxemia	218 (24.1%)	51 (16.2%)	0.56 (0.39–0.79, *P* = .001)	0.54 (0.34–0.86, *P* = .010)
	Severe hyperoxemia	257 (28.4%)	83 (26.3%)	0.77 (0.57–1.04, *P* = .086)	0.60 (0.30–1.20, *P* = .147)

Charlson = Charlson comorbidity index, Loc_icu = length of stay in ICU, LODS = logistic organ insufficiency, Los_hospitial = length of stay in hospital, OASIS = Oxford acute disease severity, RCS_PaO2 = PaO2 group in the restricted cubic spline model, RRT = renal replacement treatment, SAPS-II = simplified acute physiology score Ⅱ, SOFA = sequential organ failure assessment.

## Author contributions

**Data curation:** Anqi Wang.

**Formal analysis:** Anqi Wang.

**Investigation:** Anqi Wang.

**Methodology:** Anqi Wang.

**Resources:** Jieying Chen.

**Visualization:** Jieying Chen.

**Writing – review & editing:** Jianling Gao.
